# AlphaFamImpute: high-accuracy imputation in full-sib families from genotype-by-sequencing data

**DOI:** 10.1093/bioinformatics/btaa499

**Published:** 2020-05-28

**Authors:** Andrew Whalen, Gregor Gorjanc, John M Hickey

**Affiliations:** The Roslin Institute and Royal (Dick) School of Veterinary Studies, The University of Edinburgh, Midlothian EH25 9RG, UK

## Abstract

**Summary:**

AlphaFamImpute is an imputation package for calling, phasing and imputing genome-wide genotypes in outbred full-sib families from single nucleotide polymorphism (SNP) array and genotype-by-sequencing (GBS) data. GBS data are increasingly being used to genotype individuals, especially when SNP arrays do not exist for a population of interest. Low-coverage GBS produces data with a large number of missing or incorrect naïve genotype calls, which can be improved by identifying shared haplotype segments between full-sib individuals. Here, we present AlphaFamImpute, an algorithm specifically designed to exploit the genetic structure of full-sib families. It performs imputation using a two-step approach. In the first step, it phases and imputes parental genotypes based on the segregation states of their offspring (i.e. which pair of parental haplotypes the offspring inherited). In the second step, it phases and imputes the offspring genotypes by detecting which haplotype segments the offspring inherited from their parents. With a series of simulations, we find that AlphaFamImpute obtains high-accuracy genotypes, even when the parents are not genotyped and individuals are sequenced at <1x coverage.

**Availability and implementation:**

AlphaFamImpute is available as a Python package from the AlphaGenes website http://www.AlphaGenes.roslin.ed.ac.uk/AlphaFamImpute.

**Supplementary information:**

Supplementary data are available at *Bioinformatics* online.

## 1 Introduction

AlphaFamImpute is a software package for calling, phasing and imputing genome-wide genotypes in full-sib families when individuals are genotyped with single nucleotide polymorphism (SNP) array or genotyping-by-sequencing (GBS) data. Many applications in genetics and breeding rely on the availability of low-cost high-accuracy genotypes. GBS is an alternative to SNP arrays (Baird *et al.*, 2008; Davey *et al.*, 2011; Elshire *et al.*, 2011), where specific restriction enzymes are used to focus sequencing resources on a limited number of cut sites. GBS is particularly attractive for species without an existing SNP array or as a low-cost alternative to SNP arrays (e.g. Gorjanc *et al.*, 2015, 2017).

GBS data, and in particular low-coverage GBS data, suffer from a large proportion of missing or, when naively called, incorrect genotypes. Unlike SNP array data, where genotypes are called directly from the genotyping platform, with GBS data genotypes must be called from observed sequence reads. It is challenging to accurately call an individual’s genotype when no reads or a small number of reads are generated at a particular locus. Genotype calling accuracy can be increased by considering the haplotypes of other individuals in the population and detecting shared haplotype segments between individuals (Davies *et al.*, 2016; Gorjanc *et al.*, 2017).

Some existing software packages can be used for genotype calling and imputation from GBS data, e.g. *Beagle* (Browning and Browning, 2009), *STITCH* (Davies *et al.*, 2016), *AlphaPeel* (Whalen *et al.*, 2018) or *magicimpute* (Zheng *et al.*, 2018). However, these software packages are not designed to exploit specific structure of haplotype sharing observed in large full-sib families. As with traditional imputation methods (e.g. Antolín *et al.*, 2017; O'Connell *et al.*, 2014), we expect that the accuracy of genotype calling, phasing and imputation from GBS data is highest when population structure is taken into account. In the context of an outbred full-sib family, imputation can be simplified by recognizing that we only need to consider the four parental haplotypes and identify of which pair of haplotypes the offspring inherited at each locus. Here, we describe our software package AlphaFamImpute that leverages this particular population structure to improve the accuracy of calling, phasing and imputing genome-wide genotypes and which decreases run-time compared to existing methods. We focus on outbred full-sib families because this represents a population structure commonly found in research populations and in animal and plant breeding programs.

## 2 Materials and methods

AlphaFamImpute performs imputation using a two-step approach. In the first step, we call, phase and impute parental genotypes based on the segregation states of their offspring. Segregation states indicate which pair of parental haplotypes an individual inherits at each locus (Ferdosi *et al.*, 2014). We carry out this step iteratively. At each locus, we use the segregation states to project the offspring data to the corresponding parental haplotypes. We combine these parental haplotype estimates with the parents’ data to call parental genotypes at the locus. We then update the offspring segregation states based on the called parental genotypes. Unlike *magicimpute* (Zheng *et al.*, 2018) or *hsphase* (Ferdosi *et al.*, 2014), we do not call the segregation states of the offspring at each locus, but instead store segregation probabilities that are used to project the offspring genotypes to the parents at each locus. This allows us to account for uncertainty in the segregation states, particularly in cases where individuals have low-coverage or missing data. In the second step, we call, phase and impute the offspring genotypes by detecting which haplotype segments the offspring inherit from their parents. This process is carried out in a hidden Markov model framework using multi-locus iterative peeling (Whalen *et al.*, 2018). For a detailed description of the approach, see Supplementary Materials.

Our two-step approach builds closely on previous research. It can be interpreted as: (i) a sampling scheme for multi-locus iterative peeling (Meuwissen and Goddard, 2010; Whalen *et al.*, 2018); (ii) a probabilistic extension of *hsphase* for full-sib GBS data (Ferdosi *et al.*, 2014) or (iii) an adaptation of *magicimpute* to specifically handle low-coverage GBS data with outbred full-sib individuals (Zheng *et al.*, 2018).

## 3 Software

AlphaFamImpute is written in Python 3 using the *numpy* (Walt *et al.*, 2011) and *numba* (Lam *et al.*, 2015) libraries. It runs on Windows, Linux and Mac. As inputs, AlphaFamImpute takes in: (i) a genotype file or a sequence read count file, which, respectively, give the ordered genotypes or sequence read counts for each individual; (ii) a pedigree file which splits the population into full-sib families and (iii) an optional map file which allows AlphaFamImpute to be run on multiple chromosomes simultaneously. AlphaFamImpute outputs either called genotypes or genotype dosages.

## 4 Example

We demonstrate the performance of AlphaFamImpute on a series of simulated datasets. Each dataset consisted of 100 full-sib families with outbred parents and either 4, 8, 20, 30, 50 or 100 offspring per family. We generated parental haplotypes for 200 parents on a single 100 cM chromosome with 1000 loci using MaCS (Chen *et al.*, 2009) with an ancestral genetic history set to mimic cattle (Villa-Angulo *et al.*, 2009). We then dropped the haplotypes through the pedigree of full-sib families using AlphaSimR (Gaynor *et al.*, 2019). We generated GBS data by assuming the number of reads at each locus of an individual followed a Poisson distribution with mean equal to a coverage level of 0.5×, 1×, 2× and 5× and that there was a 0.1% sequencing error rate on a per-read basis. The parents either had no GBS data, had low-coverage GBS data at the same coverage as offspring or had high-coverage (25×) GBS data. We measured imputation accuracy as the correlation between an individual’s true genotype and their imputed genotype dosage averaged across 10 replicates of 100 full-sib families. We compared AlphaFamImpute to Beagle 4.0 (Browning and Browning, 2009) running both with default parameters.

**Fig. 1.**
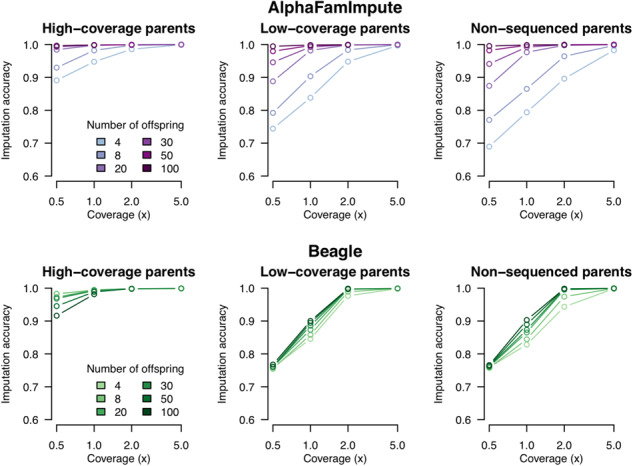
Imputation accuracy for the full-sib offspring as a function of their sequencing coverage, number of offspring and parent sequencing coverage. Results shown for both AlphaFamImpute (top) and Beagle (bottom)

Figure 1 (top) presents the imputation accuracy for all of the simulations. A more detailed analysis of the phasing and imputation accuracy is provided in Supplementary Materials. Imputation accuracy for AlphaFamImpute increased with higher GBS coverage, a larger number of genotyped offspring and more information on the parents. Imputation accuracy was high in a range of cases: if the parents were sequenced at high-coverage imputation accuracy was 0.995 with 15 offspring sequenced at 1×; if the parents were sequenced at the same coverage as the offspring, imputation accuracy was 0.990 with 10 offspring sequenced at 2× and if the parents had no data, imputation accuracy was 0.997 with 20 offspring sequenced at 2×.

The primary factor determining imputation accuracy was the total sequencing resources spent on a family. Low sequencing coverage on the parents could be compensated by sequencing additional offspring or sequencing those offspring at higher coverage. When only a few offspring were available this could be compensated by sequencing those offspring at higher coverage. Imputation accuracy may also be affected by the total number of loci sequenced.

Compared to Beagle, Figure 1 (bottom), the imputation accuracy of AlphaFamImpute was higher when the parents were sequenced at low-coverage or were not sequenced. When the parents were not sequenced, and 20 offspring were sequenced at 0.5×, the imputation accuracy of AlphaFamImpute was 0.87, while the imputation accuracy of Beagle was 0.76.

The computational requirements of AlphaFamImpute were low. When imputing 100 full-sib families with 100 offspring each (total 200 parents and 10,000 offspring) AlphaFamImpute took 54 s and used 302 megabytes of memory for 1000 loci on one chromosome. In comparison, Beagle took 11 h and used 284 megabytes of memory.

## 5 Conclusion

In this paper, we have described the AlphaFamImpute software package for performing fast, high-accuracy calling, phasing and imputing genome-wide genotypes in full-sib families from GBS data. This program will improve the quality of genome-wide genotypes from low-coverage GBS in a range of research and breeding applications.

## Supplementary Material

btaa499_Supplementary_Information
