# Supplementary material for: AlphaFamImpute: high-accuracy imputation in full-sib families from genotype-by-sequencing data
Source: Bioinformatics. 2020 May 28;36(15):4369–71. doi: 10.1093/bioinformatics/btaa499 (PMC7520044; doi:10.1093/bioinformatics/btaa499)
Supplement: btaa499_Supplementary_Information [file btaa499_supplementary_information.docx]

Supplementary Information for “AlphaFamImpute: high accuracy imputation in full-sib families from genotype-by-sequencing data”

Andrew Whalen, Gregor Gorjanc, and John M Hickey

The Roslin Institute and Royal (Dick) School of Veterinary Studies, The University of Edinburgh, Midlothian, Scotland, UK

Table of Contents

[Method description 2](#_Toc35941674)

[Genotype probabilities from read counts 2](#_Toc35941675)

[Transmission probabilities 3](#_Toc35941676)

[Calling, phasing, calling, and imputing the parental genotypes 4](#_Toc35941677)

[Calling the parental genotypes 4](#_Toc35941678)

[Updating the offspring segregation probabilities 5](#_Toc35941679)

[Additional details 6](#_Toc35941680)

[Phasing and imputing the offspring 7](#_Toc35941681)

[Accuracy on parents and offspring 8](#_Toc35941682)

[References 10](#_Toc35941683)

# Method description

AlphaFamImpute calls, phases and imputes genome-wide genotypes in full-sib families from SNP array and Genotyping-By-Sequencing (GBS) data with a two-stage approach. In the first step it phases and imputes parental genotypes based on the segregation states of their offspring – states which represent which pair of parental haplotypes the offspring inherited. In the second step it calls, phases, and imputes the offspring genotypes by detecting which haplotypes segments the offspring inherit from their parents. In the following we describe generating genotype probabilities from read-count data, the segregation transmission model and the AlphaFamImpute algorithm for calling, phasing and imputing parent and offspring.

## Genotype probabilities from read counts

In this algorithm we consider diploid individuals genotyped at bi-allelic SNP sites. This results in four possible phased genotype states, aa, aA, Aa, and AA, where ‘a’ represents a copy of the reference allele, ‘A’ represents a copy of the alternative allele, the first allele is inherited from the father, and the second allele is inherited from the mother.

With GBS data we assume the input data is sequence read counts for the reference and alternative alleles at each locus of an individual. We follow past work (Xie et al., 2010; Whalen et al., 2018) to translate the observed sequence read counts into genotype probabilities. The probability that an individual, *x*, has genotype $g_{x,i}$ at locus *i* conditional on observed genetic data $d_{x,i}$ is given by:

where $n_{ref}$ is the number of sequence reads observed for the reference allele, $n_{alt}$ is the number of sequence reads observed for the alternative allele, and $e$ is a small sequencing error rate on a per sequence read basis, assumed to be 0.1% (although it can be changed as a run-time parameter). This equation is derived by assuming that for homozygous loci each read has an independent error rate, and for heterozygous loci that reads are generated from each allele with equal probability. We also adopt the analogous equations from Whalen et al. (2018) for individuals genotyped with SNP array data.

Figure S1: Phased genotypes for a parent-offspring trio. The colors indicate parent haplotypes. In this figure the offspring inherits both grandpaternal (p) and grandmaternal (m) haplotypes from both parents. The “seg” row gives the corresponding segregation state for both of the offspring haplotypes.

If an individual is not genotyped, the genotype probability values for the individual are set to the prior distribution that the four phased genotype states are equally likely, i.e., $p\left( g_{x,i} | d_{x,i} \right)=0.25$

## Transmission probabilities

As part of the imputation and phasing algorithms, we track the segregation states for each child. These states represent which pair of parental haplotypes the individual inherits at each locus. We consider four possible segregation states: *pp*, *pm*, *mp*, and *mm*, where the first letter indicates the haplotype the offspring inherited from their father (either the father’s paternal (*p*) or father’s maternal (*m*) haplotype), and the second letter indicates the haplotype the offspring inherited from their mother. Figure S1 shows haplotypes and segregation states for a parent-offspring trio across 10 loci.

We assume that the segregation states follow a Markov process, with independent recombinations in the maternal and paternal chromosomes (Meuwissen and Goddard, 2010; Whalen et al., 2018). The probability of the segregation state of an offspring, *o*, at locus *i* conditional on the segregation state at the previous locus, *i+1* is given by:

where $\gamma$ is the per-locus recombination rate, and is the number of recombinations required to move between state $s$ and state $s^{'}$. For example, if a single recombination occurs between s and s’ then:

$$p\left( seg_{o,i}=pp | seg_{o,i+1}=pm \right)=\left( 1-\gamma\right)\gamma.$$

# Calling, phasing, calling, and imputing the parental genotypes

We call, phase and impute the genotypes of the parents using their genotype probabilities and the genotype and segregation probabilities of their offspring. We start from the last locus on the chromosome and proceed locus-by-locus until the first locus on the chromosome. At each locus we call the genotypes of the parents and re-estimate the segregation probabilities of the offspring based on the called parental genotypes. We describe these steps below.

## Calling the parental genotypes

We estimate the parental genotypes as the combination of the observed genetic information on the parents with observed genetic information of the offspring. To do this, we first calculate a “posterior” probability term for each offspring (Elston and Stewart, 1971; Kerr and Kinghorn, 1996) which gives the joint parental genotype probabilities of the parents ($g_{f,i}, g_{m,i})$ conditional on the genotype value and the segregation state of an offspring,$o$:

The value, $p\left( g_{o,i} | g_{f,i}, g_{m,i},seg_{o,i} \right),$ is the probability that an offspring has genotype $g_{o,i}$ conditional on their segregation state, and the genotypes of their parents. This value will either be 0 or 1. As an example, if the segregation state of the offspring is *mm*, where the offspring inherits both of their parents’ maternal alleles then:

$$p\left( g_{o,i}=aa | g_{f,i}=Aa, g_{m.,i}=aa,seg_{o,i}=mm \right)=1.$$

Due to possible uncertainty about the underlying genotype state of the offspring and their segregation state, we marginalize over the genotype probabilities for the offspring, $p\left( g_{o,i} | d_{o,i} \right)$ (calculated from Equation 1), and segregation probabilities, $p\left( seg_{o, i} | seg_{o,i+1} \right)$ (calculated from Equation 2). This means that the posterior term at each locus depends on the segregation values at the previous loci.

Once the posterior terms are calculated, we evaluate the joint parental genotype probabilities by combining the parent’s own genetic information, $p\left( g_{f,i} | d_{f,i} \right)$ and $p\left( g_{m,i} | d_{m,i} \right)$, with the genetic information from each offspring:

We called genotypes, $g_{m,i}^{*}$ and $g_{f,i}^{*}$, using:

If there are multiple genotype states that satisfy Equation 5, then we select one of those states uniformly at random.

## Updating the offspring segregation probabilities

Once we call parental genotypes at a particular locus, we update the offspring segregation probabilities. We do this update by combining information from the right-hand segregation probability, $g(seg_{o,i}, seg_{o,i+1})$, with the conditional probability of an individual’s segregation state conditional on the parental genotypes, $f\left( seg_{o,i},g_{m,i}^{*},g_{f,i}^{*} \right)$:

For the first term, the probability of an individual’s segregation state conditioned on the genotype state of their parents is:

where $p\left( g_{o,i} | g_{m,i}^{*},g_{f,i}^{*},seg_{o,i} \right)$ is as in Equation 3, and $p\left( g_{o,i} | d_{o,i} \right)$ is given by Equation 1. For the second term, we calculate the probability of the segregation state at the current locus based on the segregation state at the previous locus,

$$g\left( seg_{o,i}, seg_{o,i+1} \right)=\sum_{seg_{o,i+1}} p\left( seg_{o,i} | seg_{o,i+1} \right) p\left( seg_{o,i+1} \right|g_{m,i+1}^{*},g_{f,i+1}^{*})$$

where $p\left( seg_{o, i} | seg_{o,i+1} \right)$ is given by Equation 2. To account for uncertainty in the segregation sates, we marginalize over the segregation state at the previous locus.

## Additional details

We made two minor modifications to this approach in order to increase accuracy. First, we phase the parental genotypes twice: in a backward pass where the initial segregation probabilities are set to a uniform value, $p\left( seg_{o,N} \right)=.25$ (where N is the number of loci), and in a forward pass where the segregation probabilities at the first locus are based on the final segregation probabilities from the backward pass:

where the asterisk (*) denotes that this is the probability for the forward pass. Working in a two-pass approach allows us to correctly handle uncertainty in the segregation states at the start of the backward pass, while still maintaining high accuracy at both ends of the chromosome. Second, we avoid underflow in Equation 1 and Equation 4 by calculating and combining the genotype probability estimates on a log-scale, as done by Kerr and Kinghorn (1996), for example.

# Phasing and imputing the offspring

We use multi-locus peeling to call, impute, and phase the offspring. This method is equivalent to using a diploid hidden Markov model where the parental haplotypes form the reference libraries for each of the offspring’s haplotypes (Li and Stephens, 2003). We calculate the probability of an individual’s genotype based on their own genetic data, and the called parental genotypes conditional on the individual’s segregation probabilities:

where $p\left( g_{o,i} | d_{o,i} \right)$ is given by Equation 1 and we calculate the left-hand and right-hand segregation probabilities, $p\left( seg_{o,i} | seg_{o,i+1} \right)$ and $p(seg_{o,i}|seg_{o,i-1})$, recursively using Equation 8.

Based on past experience, we have found that incorrectly called parental genotypes can reduce imputation accuracy. In order to mitigate this issue, we only evaluate the segregation probabilities in Equation 8 based on loci where $p\left( g_{f,i}^{*},g_{m,i}^{*} \right)>.95$. We also modify Equation 9 to marginalize across parental genotypes states, replacing $p\left( g_{c,i} \right|g_{m,i}^{*}, g_{f,i}^{*},seg_{c,i})$ with

# Accuracy on parents and offspring

AlphaFamImpute is able to accurately impute and phase both the parents and the offspring of full-sib families across a range of scenarios. We measured imputation accuracy as the correlation between an individual’s true genotype and their imputed genotype dosage. Phasing accuracy was measured by the switch error rate, calculated on loci which were correctly imputed as heterozygous. When the parents were sequenced at low-coverage or were not sequenced, there was a chance that the imputed genotypes of the parents will be swapped. To account for this, we evaluated the genotype and phase accuracy comparing an individual’s genotype to both the imputed genotype of themselves and their mate, and selected the higher accuracy value.

Imputation accuracy and phasing accuracies of the offspring were higher with high sequencing coverage on the parents and the offspring, and larger numbers of offspring sequenced. Imputation and phasing accuracy for the offspring are presented in Figure S1. It was possible to obtain a high, >0.99, imputation accuracy if sufficient sequencing resources were spent on the offspring, even if the parents were not sequenced. The same does not hold true for phasing. If the parents are not sequenced, there may be residual phasing errors in the offspring. This will be driven by cases where it is challenging to tell which allele came from which parent. As an example, if the parents are homozygous for opposing alleles, the algorithm will correctly impute the parents as homozygous (and impute the offspring as heterozygous) by will not be able to distinguish which parent is homozygous for which allele.

Imputation accuracy and phasing accuracy of the parents were higher with higher sequencing coverage on the parents and offspring, and larger numbers of offspring sequenced. The phasing and imputation accuracy for the parents is given in Figure S2. When the parents were sequenced at high-coverage, having a large number of offspring sequenced was not important to impute the parents, but was important to correctly phase the parents. The requirement to phase the parents likely drives the increase in offspring imputation accuracy based on increasing number of sequenced offspring. In addition, when the parents were not sequenced, the imputation accuracy of the parents never reached 1.00. This, again, is likely due to loci that are homozygous for opposing alleles. The algorithm is able to correctly assess that both parents are homozygous, but may be unable to assign the correct genotype to each parent.

Figure S1. Imputation and phasing accuracy for the offspring of full-sib families as a function of sequencing coverage, number of offspring, and parent sequencing level.

Figure S2. Imputation and phasing accuracy for the parents of the full-sib families as a function of sequencing coverage, number of offspring, and parent sequencing level.

# References

Elston, R.C., and Stewart, J. (1971). A general model for the genetic analysis of pedigree data. Hum. Hered. *21*, 523–542.

Kerr, R.J., and Kinghorn, B.P. (1996). An efficient algorithm for segregation analysis in large populations. Journal of Animal Breeding and Genetics *113*, 457–469.

Li, N., and Stephens, M. (2003). Modeling linkage disequilibrium and identifying recombination hotspots using single-nucleotide polymorphism data. Genetics *165*, 2213–2233.

Meuwissen, T., and Goddard, M. (2010). The Use of Family Relationships and Linkage Disequilibrium to Impute Phase and Missing Genotypes in Up to Whole-Genome Sequence Density Genotypic Data. Genetics *185*, 1441–1449.

Whalen, A., Ros-Freixedes, R., Wilson, D.L., Gorjanc, G., and Hickey, J.M. (2018). Hybrid peeling for fast and accurate calling, phasing, and imputation with sequence data of any coverage in pedigrees. Genetics Selection Evolution *50*, 67.

Xie, W., Feng, Q., Yu, H., Huang, X., Zhao, Q., Xing, Y., Yu, S., Han, B., and Zhang, Q. (2010). Parent-independent genotyping for constructing an ultrahigh-density linkage map based on population sequencing. Proc Natl Acad Sci USA *107*, 10578.
